# Visualizing mental representations in schizophrenia patients: A reverse correlation approach

**DOI:** 10.1016/j.scog.2019.100138

**Published:** 2019-04-06

**Authors:** Loek Brinkman, Ron Dotsch, Jelmer Zondergeld, Martijn G.J.C. Koevoets, Henk Aarts, Neeltje E.M. van Haren

**Affiliations:** a department of Psychology, Utrecht University, Utrecht, the Netherlands; b University Medical Centre Utrecht Brain Centre, dept of Psychiatry, Utrecht, The Netherlands; c Department of Child and Adolescent Psychiatry/Psychology, Erasmus Medical Centre, Rotterdam, the Netherlands

**Keywords:** Emotion recognition, Schizophrenia, Mental representations, Psychophysics, Data-driven

## Abstract

Schizophrenia patients have difficulties recognizing emotional states from faces, in particular those with negative valence, with severe consequences for daily life. What do these patients see in their minds eye, when they think of a face expressing a particular emotion or trait? The content of such mental representations can shed light into the nature of their deficits, but are usually inaccessible. For the first time, we explored the applicability of reverse correlation, which has been successfully used to visualize mental representations in healthy populations, to visualize mental representations in schizophrenia patients.

We investigated mental representations of trustworthy faces, a primary dimension of social face evaluation that is highly correlated with valence. Patients (*n* = 23) and healthy controls (*n* = 34) classified images of noise-distorted faces as ‘trustworthy’, ‘untrustworthy’ or ‘neutral’. We visualized their mental representations of these concepts by averaging the noise patterns based on their classifications. These visualizations were then rated on trustworthiness by an independent sample of participants.

Patients were able to perform the reverse correlation task, with response times and biases similar to those of healthy controls, and the obtained images vividly reflected the respective constructs of interest. However, there were no significant differences between the ratings of the visualizations of patients and controls.

Conclusion: These novel findings provide a proof of principle that the reverse correlation technique can be applied to investigate mental representations in schizophrenia patients.

## 1 Introduction

Deficits in social cognitive functioning are consistently reported in patients with schizophrenia (Green et al., 2015; Pinkham, 2014), and have been shown to be detrimental to daily functioning and quality of life (Green et al., 2005) as they undermine normal social interactions (Fett et al., 2011; Savla et al., 2013). Abnormalities in social cognition are often caused by deficits in social perception (Kohler et al., 2010). For example, schizophrenia patients have difficulties recognizing emotional expressions from faces (Kohler et al., 2010), in particular for emotions with negative valence (Kohler et al., 2003; Mandal et al., 1998). In an attempt to understand the mechanisms of poor recognition of emotions on faces, we set out to apply an innovative technique, called ‘reverse correlation’, to visualize ones' mental image of a face with a particular state or trait. What do patients with schizophrenia see in their minds eye, when they think of a fearful or trustworthy face? The content of such mental representations can provide novel insights in how social perception is distorted in these patients. However, such mental representations are often inaccessible or hard to verbalise.

**Fig. 1:**
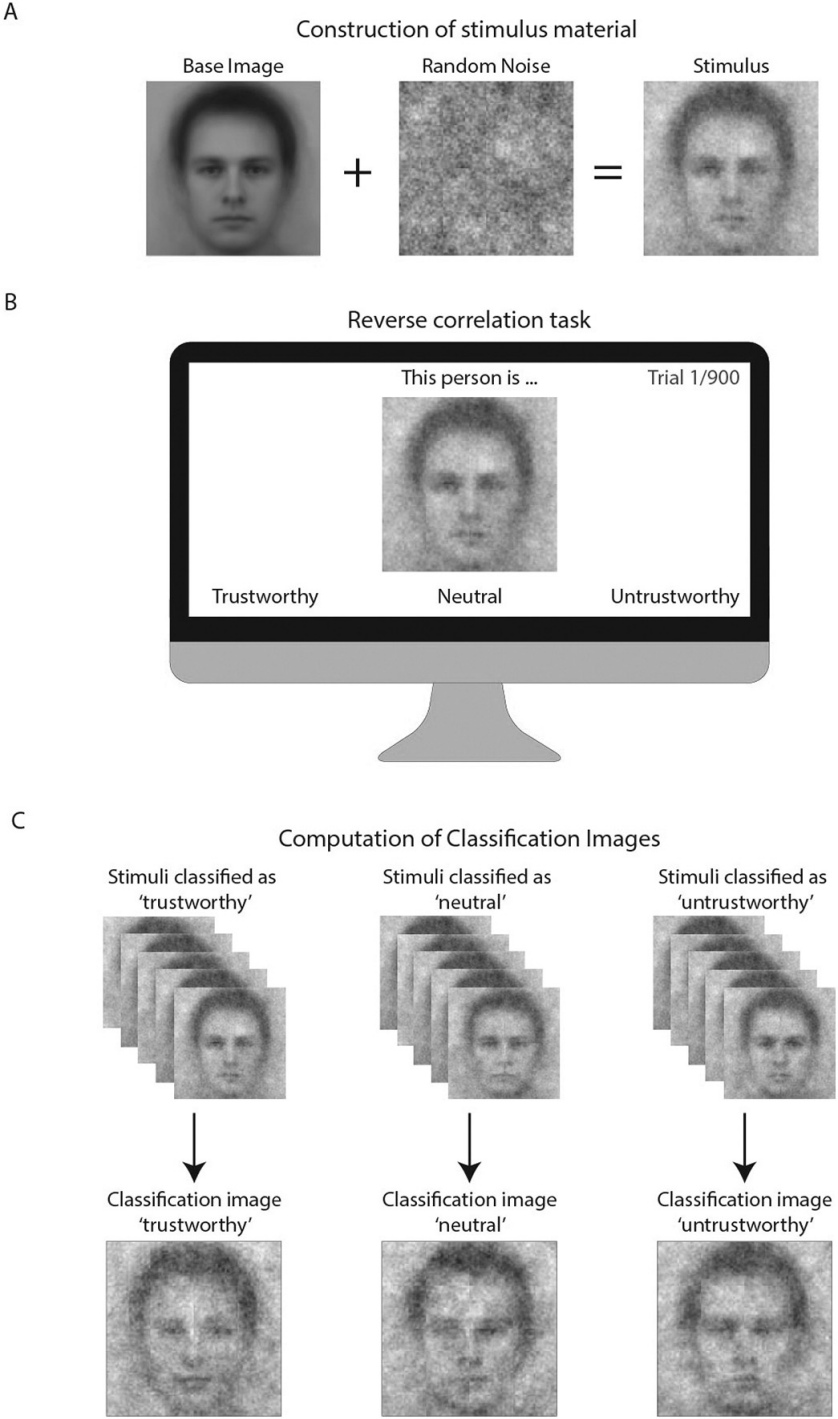
(A) Stimuli consisted of a base image of a male face with neutral expression, on which random noise patterns were superimposed. The resulting stimuli are random variations on the base image. (B) In the reverse task, one stimulus was presented at the centre of the screen which the participant classified as ‘trustworthy’, ‘neutral’ or ‘untrustworthy’. This procedure was repeated over 900 trials in total, divided over two days (450 trials on each day). (C) Classification images were computed for each of the response categories, by superimposing the average noise patterns of the selected stimuli on the base image. As such, three classification images were obtained per participant, reflecting the mental representations of a trustworthy, neutral and untrustworthy face, respectively.

Reverse correlation, a data-driven psychophysical method, has recently become popular in the field of social face perception to obtain visual read-outs of mental representations of facial appearances of states and traits (Brinkman et al., 2017; Dotsch and Todorov, 2012; Jack and Schyns, 2017; Oldmeadow et al., 2013). Participants are presented with a series of stimuli that consist of random variations of a base image. The base image is typically a face with a neutral expression. Random variations of the base image are generated by superimposing random noise patterns (Brinkman et al., 2017; Dotsch and Todorov, 2012). As each random noise pattern is different, the final stimulus set consists of slight alterations of the original base image. (Fig. 1A). The task for the participant is to classify the stimuli in a category of interest, e.g. ‘trustworthy’, ‘untrustworthy’ or ‘neutral’ (Fig. 1B). By averaging the selected random noise patterns, images are obtained that portray the features that were used for classification (Fig. 1C). These images are called ‘classification images’ (CI) and can be interpreted as visual read-outs of mental representations (Brinkman et al., 2017). In recent years, the reverse correlation method has been successfully used to visualize a plethora of mental representations, e.g. emotions (Jack et al., 2012; Mangini and Biederman, 2004), personality traits (Hehman et al., 2015; Imhoff et al., 2013), age (van Rijsbergen et al., 2014), race (Dotsch et al., 2011, Dotsch et al., 2008; Imhoff et al., 2011), professions (Hehman et al., 2015; Imhoff et al., 2013) and biases (Dotsch et al., 2008; Imhoff and Dotsch, 2013; Young et al., 2014) (reviewed in (Brinkman et al., 2017; Dotsch and Todorov, 2012; Jack and Schyns, 2017; Oldmeadow et al., 2013)).

We investigate for the first time whether the reverse correlation approach can be applied to visualize mental representations in schizophrenia patients. As the time we had to test each of our patients was limited, an elaborate exploration of deficits in each emotion was beyond the scope of this study. Instead, we focus on a primary dimension of social face perception: trustworthiness. Trustworthiness is spontaneously inferred from faces (Klapper et al., 2016), and is highly correlated with valence (Oosterhof and Todorov, 2008). As such, we reasoned that deficits the mental representation of any emotion would be reflected in the dimension of (un)trustworthiness. Trustworthiness has been extensively investigated with reverse correlation approaches in healthy populations (Oosterhof and Todorov, 2008; Sutherland et al., 2016; Todorov et al., 2015). This makes the dimension of trustworthiness a suitable starting point to evaluate the reverse correlation approach for schizophrenia patients. One needs to bear in mind that a typical reverse correlation task consists of at least a few hundred trials, making the task cognitively demanding and boring. Due to cognitive deficits, in particular in sustained attention and motivation problems (Green et al., 2015; Pinkham, 2014), applicability of such a task needs investigation for this and other patient populations. The aims of this study are therefore two-fold: (1) To test whether to reverse correlation approach can be applied schizophrenia patients, and (2) to investigate whether schizophrenia patients have different mental representations of trustworthy faces, compared to healthy controls.

## 2 Methods

### 2.1 Participants

Participants were part of the ‘Social Cognition and Imaging in Psychiatry II’ study (SCIPII) at the University Medical Centre Utrecht (UMCU), the Netherlands, where a cohort of 60 schizophrenia patients and 60 healthy controls performed a battery of tasks, over the course of two days. Patients were recruited from the UMCU psychiatry department, and local psychiatry departments in Utrecht, the Netherlands. Healthy controls were recruited via online recruitment websites and from advertisements on notice boards. Inclusion criteria were an age between 18 and 50 years old, Dutch speaking, and premorbid IQ > 80 (estimated by the Dutch Adult Reading Test (Schmand et al., 1991)). Exclusion criteria were drug- or alcohol abuse in the six months prior to testing, history of closed-head injury, neurological illness, endocrinological dysfunction, and/or chronic use of medication known to influence brain functioning. For healthy controls specifically, exclusion criteria were having (or having had) a psychiatric disorder with psychotic features, and/or having first or second-degree family members with a disorder in the psychosis spectrum. For patients only, having an acute psychotic episode at the moment of testing was an exclusion criterion. Psychiatric medication use was permitted. It should be noted here that psychiatric medication has little to no effect on facial affect recognition (Gabay et al., 2015; Hempel et al., 2010). All participants underwent screening procedures to check for the presence of exclusion criteria and signed informed consent. Participants were financially compensated for their participation. This study has been approved by the UMCU's Human Medical Ethics Committee.

The reverse correlation task was added to the task battery after the initial start of SCIPII, which is why the first participants of the cohort did not perform the task. In total, 74 participants performed the reverse correlation task. Four participants were excluded because they did not comply with task instructions. Of the remaining participants (*n* = 70), 57 participants (23 patients, 34 controls; see Table 1 for demographics) completed all trials of the reverse correlation trials, and 13 participants (9 patients, 4 controls) stopped before the end of the task, because of time constraints. The relatively high incidence of incomplete datasets amongst patients may be due to the fact that the task was the last experiment of the day of testing, so a delay in any of the preceding experiments of the SCIPII-batch (which happened more often for patients than controls) resulted in participants running short of time for the reverse correlation task. Here, we report the results of the 57 participants who completed all trials of the task. A sensitivity analysis (G*Power 3.1) for the main analysis of interest (the interaction of group (patient, control) x response category (trustworthy, neutral, untrustworthy)) showed that we had 80% power to detect a significant interaction with a partial η^2^ > 0.15 (medium to large effect size), which is the range of social cognitive deficits in schizophrenia patients (Fiszdon et al., 2013).

**Table 1:** Demographic and clinical information of patients and controls.

	Controls (*n* = 34)	Patients (*n* = 23)	Test statistics	*p*
	Mean (sd)	Mean (sd)		
Age (years)	37.58 (7.37)	37.31 (8.58)	*t* = −0.124	0.90
Range	20.16–49.42	20.58–50.92		
Sex (M/F)	33/1	22/1	χ^2^(2) = 0.000	1
Subject education (years)	14.12 (2.03)	13.43 (2.09)	*t* = 1.226	0.23
Parental education (years)	12.86 (2.69)	12.64 (3.84)	*t* = 0.229	0.82
Premorbid IQ	101.74 (8.31)	97.69 (10.73)	*t* = 1.523	0.14
PANSS total		52.35 (11.25)		
Positive		13.64 (4.28)		
Negative		13.48 (4.01)		
General		25.22 (5.85)		
Illness duration (years)		17.01 (9.45)		
Medication type				
Typical		3 (13.04		
Atypical		20 (86.96)		

Additionally, we provide the results of all participants, including those with incomplete datasets, in the Supplementary Material (*n* = 70; 32 patients, 38 controls). Inclusion of incomplete datasets did not alter any of the main findings.

### 2.2 Positive and Negative Syndrome Scale (PANSS)

The ‘Positive and Negative Syndrome Scale’ (PANSS) was administered by trained psychologists to evaluate severity of current symptoms (Kay et al., 1987). The PANSS is a 30-item rating scale with three subscales, representing positive, negative, and general psychopathology symptoms. Each symptom is rated on a seven-point scale (1 = absent to 7 = extremely severe).

### 2.3 Procedure & reverse correlation task

Participants were seated in a closed room and performed the reverse correlation task on a desktop computer. Task instructions were given both on screen and verbally by the task leader. Participants performed 900 trials of the reverse correlation task. The task was divided over two days to reduce the load on the participant (450 trials on each day) with breaks every 75 trials. On each trial, a noisy image of a face was presented at the centre of the screen (512 × 512 pixels, presented at 15.6 × 14.04 cm; Fig. 1B). Participants classified the faces as ‘trustworthy’, ‘neutral’ or ‘untrustworthy’, using the respective response keys on the keyboard (A, spacebar, L) as indicated on the bottom of the screen (three-option forced choice). Original data was acquired and stored on a custom server. Due to privacy issues, the original data is not publicly available. We are currently investigating which anonymized parts of the data can be made public, which we will make available as soon as possible.

The noisy faces were constructed by superimposing a random noise pattern on a base image. The base image was a male Caucasian face with a neutral facial expression, which was the average of all male faces with neutral expression of the Karolinska face database (Lundqvist and Litton, 1998). For each stimulus, a different random noise pattern was superimposed on the base image. The random noise patterns consisted of combinations of sinusoidal noise patches of different angles, spatial frequencies, phases and amplitudes. Random noise patterns were obtained by randomizing the amplitude of the sine-waves that determine the noise pattern. This procedure is explained in detail elsewhere (Dotsch and Todorov, 2012) and follows the default setting in the ‘rcicr’ R-package (Dotsch, 2017), which contains the code to create the stimuli. This procedure yields noise patterns that vary in the frequency spectrum of facial features, which lead to stimuli that are variations of the base image (Mangini and Biederman, 2004). Base image, stimuli, and R-code to construct these stimuli are available on the Open Science Framework (https://osf.io/zujpe/).

It should be noted here that there are many variants of the reverse correlation method, e.g. those that use 3D computer generated faces or photo's instead of noise-based images (Jack and Schyns, 2017; Oldmeadow et al., 2013) and/or vary in the number of response options (Brinkman et al., 2017). We chose the noise-based reverse correlation task with three response options, as it is relatively unconstrained in terms of stimulus variability and allows the participant to choose the ‘neutral’ response option when they are less certain. The number of trials in the experiment (two sessions of 450 trials) was a trade-off between feasibility (attention span and time available to test patients) and sensitivity. Studies that use noise-based reverse correlation task typically use 500 to 1000 trials (Brinkman et al., 2017).

### 2.4 Computation of classification images

For each participant, classification images were obtained by averaging, per response category, the noise patterns of the selected stimuli. The averaged noise patterns were rescaled to the minimal and maximal pixel values of the base image, after which the noise pattern and the base image were averaged. This procedure is explained in detail elsewhere (Dotsch and Todorov, 2012) and follows the default setting in the ‘rcicr’ R-package (Dotsch, 2017) to compute the classification images. The R-code used to compute the classification images is available on the Open Science Framework (https://osf.io/zujpe/). Fifty-two participants used all three response options during the reverse correlation task, which yielded 156 classification images. The remaining five participants (all controls) only used two response categories, which yielded an additional 10 classification images (166 images in total).

### 2.5 Rating of classification images

To evaluate the classification images, an independent sample of participants was recruited who rated the classification images on trustworthiness. Twenty-one Caucasian participants were recruited from various countries via the online participant platform Prolific (www.prolific.ac). These participants completed an online rating task where they rated classification images on trustworthiness by using a continuous slider (0–100 points) ranging from untrustworthy (left) to trustworthy (right). The rating task included 166 classification images of the 57 participants who completed all trials of the reverse correlation task as well as the 39 classification images of the 13 participants who that did not complete all trials of the reverse correlation task (205 images in total). The primary outcome measure of the rating task was the average rating per classification image.

Two raters, who had unlikely fast response times (median response time < 500 ms) were excluded from the sample, resulting in a total sample of 19 raters (11 males, 8 females, mean age ± SD: 28.4 ± 8.2). The consistency amongst the 19 raters was high (Cronbach's alpha: 0.92). The rating task was constructed on the online experiment platform Gorilla (www.gorilla.sc).

### 2.6 Analysis

We first assessed whether task performance was similar for patients and controls, comparing response times and response biases across samples. Response times were compared with a non-parametric *t*-test. Response biases were quantified as the frequency by which each of the three response options was chosen and were tested with a 2 (group: patient, control) × 3 (response category: trustworthy, neutral, untrustworthy) repeated measures ANOVA. Note that in this model, the response frequencies of two response categories (e.g. trustworthy and neutral) fully determine the response frequency of the last category (e.g. untrustworthy). Further investigation performing three 2 (group) × 2 (response categories) ANOVAs (dropping respectively the trustworthy, neutral or untrustworthy response frequency) did not yield different results.

Next, we compared trustworthiness ratings of classification images, as assessed by the independent sample of raters, with a 2 (group: patient, control) × 3 (classification image: trustworthy, neutral, untrustworthy) repeated measures ANOVA. Post-hoc *t*-tests were corrected for multiple comparisons (Bonferroni).

**Fig. 2:**
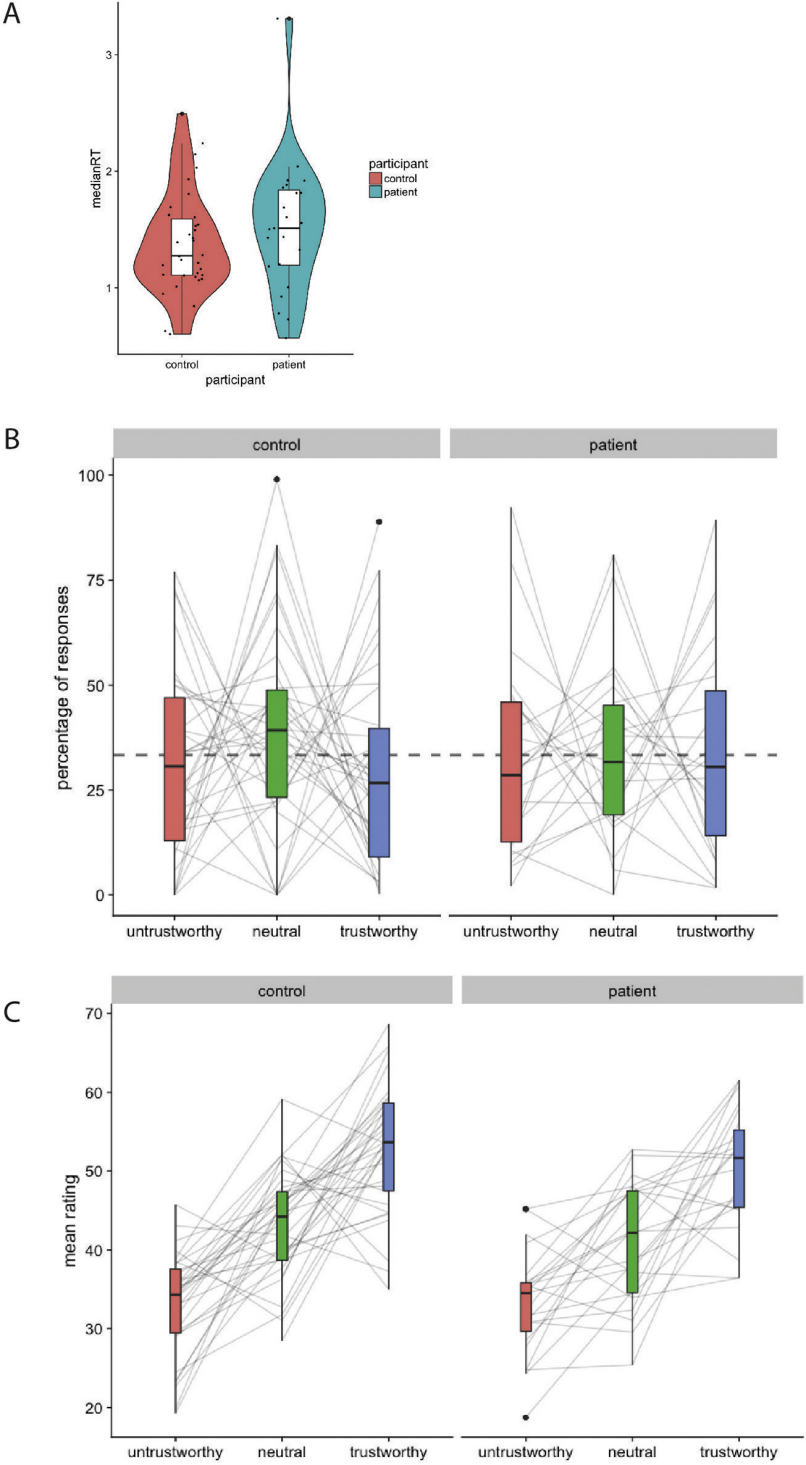
(A) Median response times per participant, for patients (right, light blue) and controls (left, ref). Dots reflect median response times for individual participants, with random jitter on the x-axis, to increase visibility of individual dots. Outlines of the violin plots represent the density distributions of the samples and are overlaid with boxplots. (B) Percentage of responses for each of the three response categories (untrustworthy: red; neutral: green; trustworthy: blue), for controls and patients (left and right panels, respectively). Grey lines represent the percentage of responses for individual participants. Dashed horizontal line represents a uniform distribution of responses (33%). (C) Subjective ratings of classification images on trustworthiness (scale 1–100) by a sample of independent raters, for controls (left panel) and patients (right panel). Colours depict the response category of the classification image (untrustworthy: red, neutral: green, trustworthy: blue). Grey lines represent the ratings of individual classification images.

More distinct mental representations of trustworthy and untrustworthy faces are expected to correlate with less severe symptoms (negative correlation). Therefore, in patients only, additional analyses were performed to investigate whether individual performance on the reverse correlation task correlated with severity of symptoms (PANSS total and subscales). Performance on the reverse correlation task was quantified as the difference between the ratings of the trustworthy and untrustworthy classification images (trustworthy - untrustworthy). This metric captures how distinct the classification images of these opposing concepts are, and represent the slope of the lines in Fig. 2C. If one of the response categories was not used by a participant, the slope was computed using the neutral condition as a reference point.

## 3 Results

Patients (*n* = 23) did not differ significantly from healthy controls (*n* = 34) in age, sex, subject education, parental education or premorbid IQ (see Table 1). Patients had a mean PANSS score of 52.35 ± 11.25 (mean ± SD), corresponding to mild impairments (Leucht et al., 2005).

### 3.1 Response times

Patients and controls did not differ significantly in response times during the reverse correlation task (W = 321, *p* = 0.26, *r* = 0.15, Mann-Whitney *U* test). The groups had similar distributions of median response times, with grand average median response times ± SD of 1.38 ± 0.44 s for controls and 1.52 ± 0.57 s for patients (Fig. 2A).

### 3.2 Response bias

There were also no indications of differences in response bias (Fig. 2B; Controls: trustworthy: 30% ± 23%; neutral: 39% ± 24%; untrustworthy: 31% ± 22%; Patients: trustworthy: 34% ± 25%; neutral: 34% ± 21%; untrustworthy: 32% ± 23% (mean-% ± SD)). A repeated measures ANOVA of group (patients, controls) and response category (trustworthy, neutral, untrustworthy) did not reveal a significant main effect of group (F(1, 55) < 0.001, *p* = 1.0, partial η^2^ = 0.01) nor a significant interaction of group and response category (F(2, 110) = 0.417, *p* = 0.66, partial η^2^ = 0.01). No consistent bias was observed amongst response categories (main effect of response category: F(2, 110) = 0.527, *p* = 0.59, partial η^2^ = 0.01). In both patient and control groups, some participants used one of the response options more often than the other two, but in those cases, it varied across participants which of the response options was chosen predominantly.

### 3.3 Independent raters

**Fig. 3:**
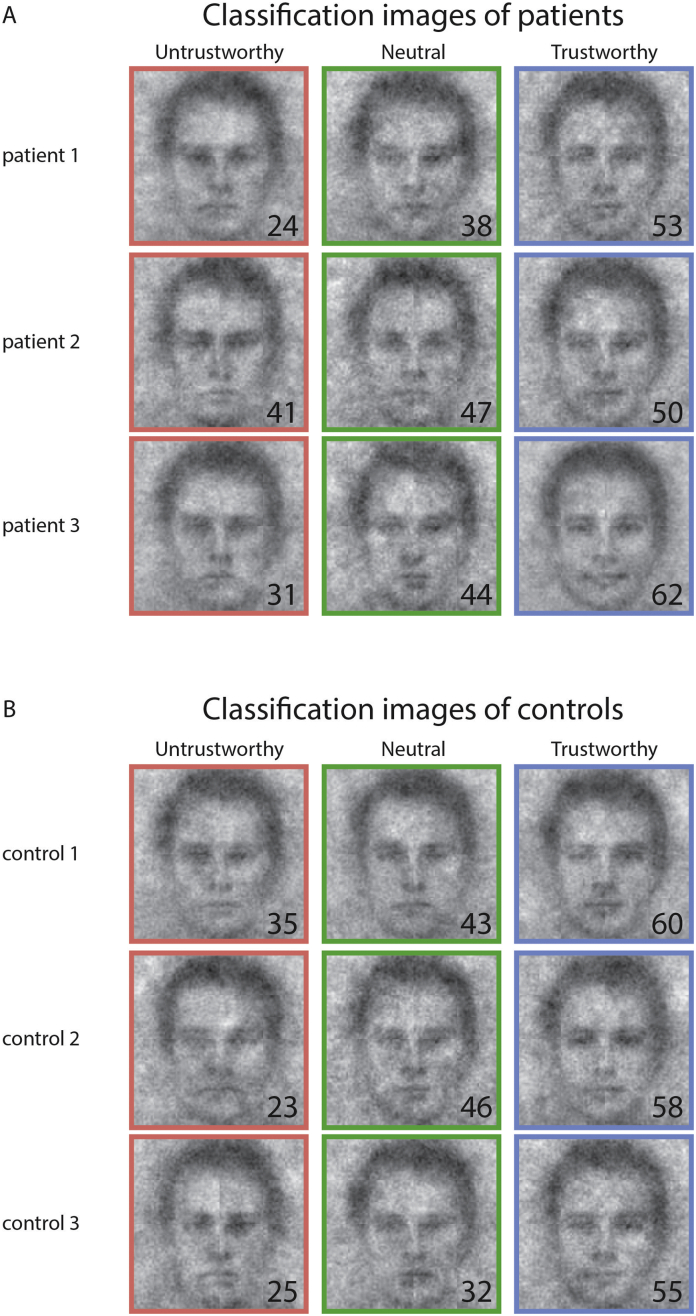
Representative classification images for patients (panel A) and controls (panel B) reflecting trustworthy, neutral and untrustworthy faces. Colours depict the response category of the classification image (untrustworthy: red, neutral: green, trustworthy: blue). The insets in the bottom-right corner depict the average rating on trustworthiness (scale 1–100) by a sample of independent raters.

All 166 images were rated on trustworthiness (scale from 1 to 100) by an independent sample of raters (*n* = 19). Fig. 2 depicts the classification images of three patients (Fig. 3A) and three control subjects (Fig. 3B), for each of the three response categories, where the average trustworthiness ratings are depicted as insets (bottom-right corner of each classification image).

The classification images of the three response categories differed strongly in trustworthiness ratings (main effect of classification image: F(2, 100) = 68.99, *p* < 0.001, partial η^2^ = 0.58). The mean ratings of the classification image of the patient group were: 50.2 ± 7.5, 40.8 ± 7.7 and 33.2 ± 6.7, for classification images reflecting trustworthy, neutral and untrustworthy faces, respectively (mean rating ± SD). For controls, these ratings were 53.0 ± 8.1, 43.0 ± 7.0 and 33.1 ± 6.6), respectively. Post-hoc *t*-test showed significant differences between classification images of trustworthy, neutral and untrustworthy faces, for all within-group comparisons (all *p*'s < 0.001, see Supplemental table 1, top and middle rows). There were no indications that ratings differed for classification images of patients and controls, neither as a main effect of group: (F(1, 50) = 2.950, *p* = 0.10, partial η^2^ = 0.056) nor as an interaction of group and classification image (F(2,100) = 0.661, *p* = 0.09, partial η^2^ = 0.01). Post-hoc *t*-tests comparing the classification images of each of the three categories (trustworthy, neutral, untrustworthy) of patients and controls did also not indicate these images to differ between groups (all *p*'s > 0.5, see Supplemental table 1, bottom rows).

### 3.4 Symptoms

Individual performance on the reverse correlation task was quantified as the difference in subjective ratings of the individual's trustworthy and untrustworthy CIs. This quantifies how distinct these two concepts are from each other, as reflected in a participant's CIs, and can be seen as the slope of the grey lines in Fig. 2C.

**Fig. 4:**
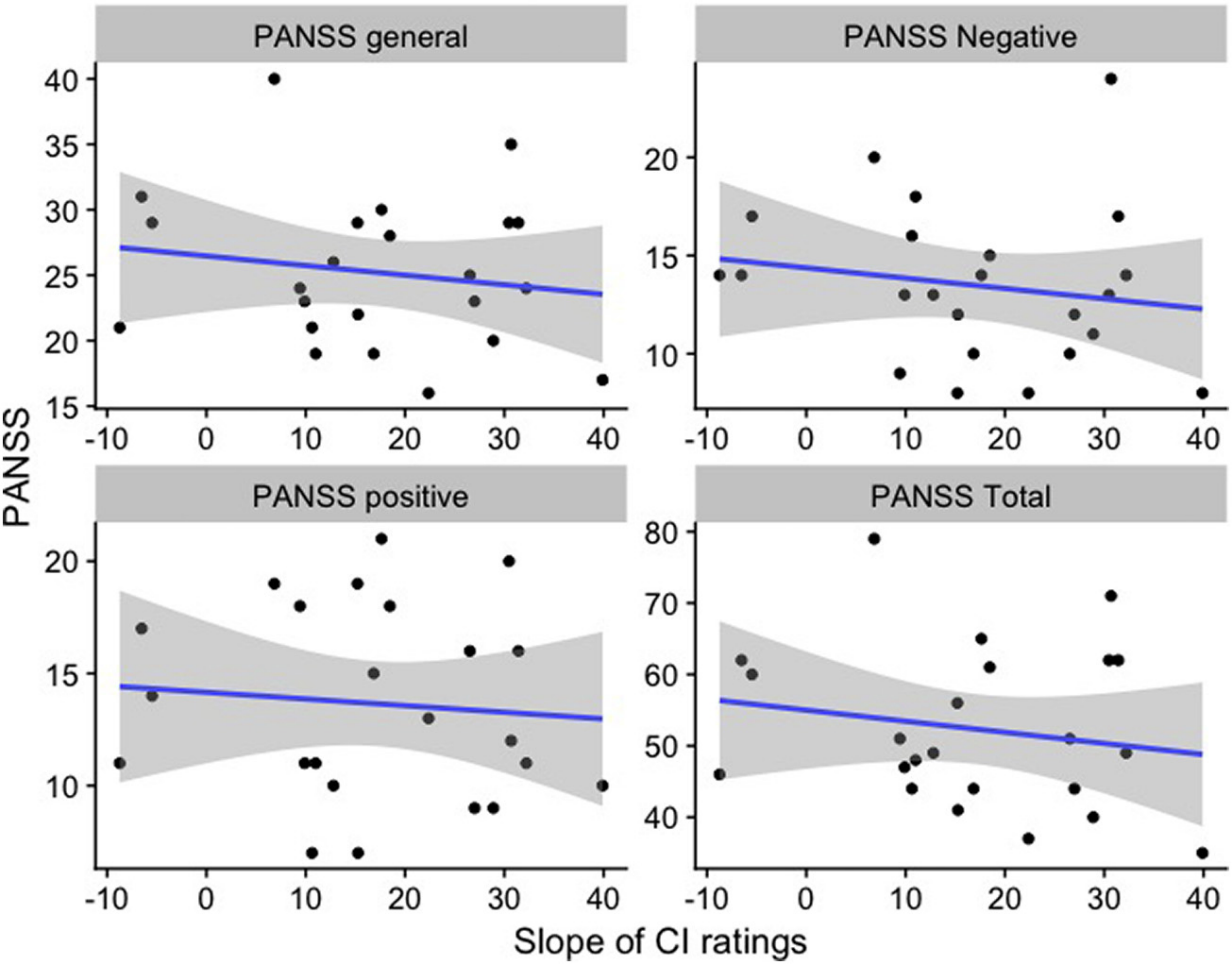
The relation of the distinctness of the classification images, quantified as the slope of the ratings of the classicisation images (grey lines in Fig. 3) and severity of symptoms (PANSS), for the total PANSS score (right-bottom) and the three PANSS subscales (PANSS general: left-top, PANSS Negative: right-top, PANSS Positive: left-bottom). Dots represent PANSS scores of individual patients. Blue horizontal lines are the linear fits (regression line). Grey shades represent standard deviations (smoothed with locally estimated scatterplot smoothing).

Although the correlations are in the expected direction (Fig. 4), where higher slopes of the ratings of the CIs corresponded to lower PANSS scores, the individual differences amongst patients are too large to detect a significant correlation with the current sample-size, for either the total PANSS score (*r* = −0.18, *p* = 0.41) or any of the PANSS sub-scores (PANSS general: *r* = −0.16, *p* = 0.46; PANSS positive: *r* = −0.09, *p* = 0.68; PANSS negative: *r* = −0.17, *p* = 0.44).

## 4 Discussion

We explored (1) whether the reverse correlation method can be applied to schizophrenia patients, and (2) whether the mental representations of trustworthy and untrustworthy faces are deviant in schizophrenia patients.

Patients were able to understand and comply with task instructions, and performed the task with no notable differences in response times or biases compared to healthy controls, providing a proof of principle that the reverse correlation approach can be applied to study visual proxies of mental representations in schizophrenia patients.

We did not observe significant differences in the online ratings of the classification images of patients and control, nor did these ratings correlate with the severity of symptoms. As such, we find no evidence for deviant mental representation of (un)trustworthy faces in schizophrenia patients. It is therefore possible that the mental representations of (untrustworthy) in schizophrenia patients faces are intact. However, it should be noted that this cannot be concluded based on this data: absence of evidence is not evidence of absence. But if the mental representations of (un)trustworthy faces are intact in schizophrenia patients, it is unlikely that these mental representations underlie their social cognitive deficits. It is possible that these deficits rely on mental representation that are more specific, e.g. to particular emotions. Follow-up studies can use the reverse correlation method to obtain visual read-outs of mental representations of particular emotions to investigate whether the level of displayed emotion in the classification images correlates with deficits in recognizing particular emotions.

In generalizing these observations to a larger population of patients, it must be noted that the symptoms of the patients in our sample were only mild. Whether patients with more severe symptoms or specific social cognitive abnormalities have deviant mental representation of trustworthy faces is open for empirical investigation.

The current findings show that the reverse correlation method can be applied to schizophrenia patients, but further research is needed to optimise the procedure. For example, patients performed two sessions of 450 trials, which was demanding and time consuming (about 20 min per sessions). It is possible that classification images of similar quality can be obtained with less trials. As seen in the supplemental material, the ratings of the classification images of participants with incomplete datasets were often quite similar to those of participants that completed all trials, as long as participants had performed at least ~700 trials. Optimizing the number of trials is one of the manners in which the current approach can be improved, albeit that the optimal number of trials likely differs from one mental representation to the other (e.g. a trustworthy face versus a fearful face). Moreover, we only investigated the applicability of one particular variant of the reverse correlation method, using noise-based stimuli and a three response-options forced choice task. We chose this particular variant, as it is relatively unconstrained in terms of stimulus variability and allows participant to choose the ‘neutral’ response option when they are less certain. Other variants of the task are available that use 3D computer generated faces or photo's (Jack and Schyns, 2017; Oldmeadow et al., 2013) and/or different numbers of response options (Brinkman et al., 2017) and it is worthwhile to consider the applicability of these task variants as well.

Regardless of the instantiation of the task, fleshing out aberrations in specific mental representations can prove to be a versatile approach to better understand the social world of schizophrenia patients. It allows researchers and clinicians a peek into the mental world of their patients. This can provide insight in the mental state and deficits of individual patients.

The current study provides a proof of principle that the reverse correlation approach can be adopted to investigate mental representations in schizophrenia patients. It is to be expected that the reverse correlation method can also be applied to investigate mental representations in other mental illnesses where mental representations may be compromised, such as anorexia nervosa (Gadsby, 2017), depression (Chekroud, 2015) and psychosomatic disorders (Edwards et al., 2012).

## Conflict of interest

The authors have declared that there are no conflicts of interest in relation to the subject of this study.

## Funding

This work was supported by a grant from the Netherlands organization for scientific research: NWO-VIDI452-11-014 (NEM van Haren).

## Contributions

Author LB designed the study, analysed the data and wrote the manuscript. Authors NEMvH, HA and RD designed the study and wrote the manuscript. Authors JZ and MGJCK collected the data.

## Appendix A Supplementary data

Supplementary material Image 1
