# Supplementary material for: Visualizing mental representations in schizophrenia patients: A reverse correlation approach
Source: Schizophr Res Cogn. 2019 Apr 6;17:100138. doi: 10.1016/j.scog.2019.100138 (PMC6454059; doi:10.1016/j.scog.2019.100138)
Supplement: Supplementary file 1 — Supplementary material [file mmc1.docx]

**Supplemental material**

| **Classification image A** | **Classification image B** | **df** | **t** | **p**  **(Bonf. corrected** | **Effect-size (d)** | **95% conf. interv. on d**  **(lower)** | **95% conf. interv. on d**  **(upper)** |
| --- | --- | --- | --- | --- | --- | --- | --- |
| Trust - control | Untrust- control | 62.8 | 11.0 | < .001 | 2.69 | 2.01 | 3.36 |
| Trust - control | Neutral- control | 62.8 | 5.36 | .001 | 1.32 | 0.78 | 1.87 |
| Neutral- control | Untrust - control | 60.5 | 8.15 | .008 | 1.45 | 0.89 | 2.02 |
|  |  |  |  |  |  |  |  |
| Trust - patient | Untrust - patient | 43.3 | 8.15 | < .001 | 2.40 | 1.62 | 3.18 |
| Trust - patient | Neutral - patient | 44.0 | 4.24 | < .001 | 1.25 | 0.60 | 1.9 |
| Neutral - patient | Untrust - patient | 43.1 | 3.59 | < .001 | 1.06 | 0.42 | 1.69 |
|  |  |  |  |  |  |  |  |
| Trust - control | Trust - patient | 49.6 | 1.3 | 1.0 | 0.35 | -0.20 | 0.89 |
| Untrust - control | Untrust -patient | 45.0 | 1.08 | 1.0 | 0.30 | -0.25 | 0.86 |
| Neutral - control | Neutral - patient | 47.4 | -0.05 | 1.0 | -0.01 | -0.56 | 0.54 |

**Supplemental Table 1 – table of post-hoc *t*-tests related to Figure 3 of the main text:** relevant post-hoc Welch t-tests of the repeated measures ANOVA of participant group (patients, controls) and trustworthiness rating (trustworthy, neutral, untrustworthy). Classification image A and B represent the two categories of classification images that are compared in the t-test. ‘Trust’, ‘neutra’l and ‘untrust’ reflect the classification images of trustworthy, neutral and untrustworthy faces, respectively. Top rows: comparison of response categories within controls; middle rows: comparison of response categories within patients; bottom rows: comparison of response categories between patients and controls.

|  | **Controls (n=38)** | **Patients (n=32)** | **Test statistics** | **p** |
| --- | --- | --- | --- | --- |
|  | Mean (sd) | Mean (sd) |  |  |
|  |  |  |  |  |
| Age  (years) | 38.08 (7.45) | 36.88 (8.35) | t = 0.632 | .53 |
| Range | 20.17 - 50.40 | 20.58 - 50.92 |  |  |
| Sex  (M/F) | 37/1 | 30/2 | χ2(2) =0.02 | .88 |
| Subject education (years) | 14.00 (2.03) | 13.35 (1.98) | t = 0.188 | .32 |
| Parental education (years) | 12.72 (2.64) | 13.10 (3.63) | t = 0.636 | .12 |
| Premorbid IQ | 101.82 (8.26) | 98.15 (9.71) | t = 1.661 | .10 |
| PANSS Total |  | 51.10 (10.50) |  |  |
| Positive |  | 13.16 (3.87) |  |  |
| Negative |  | 12.97 (4.35) |  |  |
| General |  | 24.00 (5.24) |  |  |
| Illness duration (years) |  | 15.97 (8.80) |  |  |
| Medication Type |  |  |  |  |
| Typical  Atypical |  | 3 (9.4)    29 (90.6) |  |  |
|  |  |  |  |  |

**Supplemental Table 2**: demographics of patients and controls (n = 70), including those with incomplete datasets (n = 13).


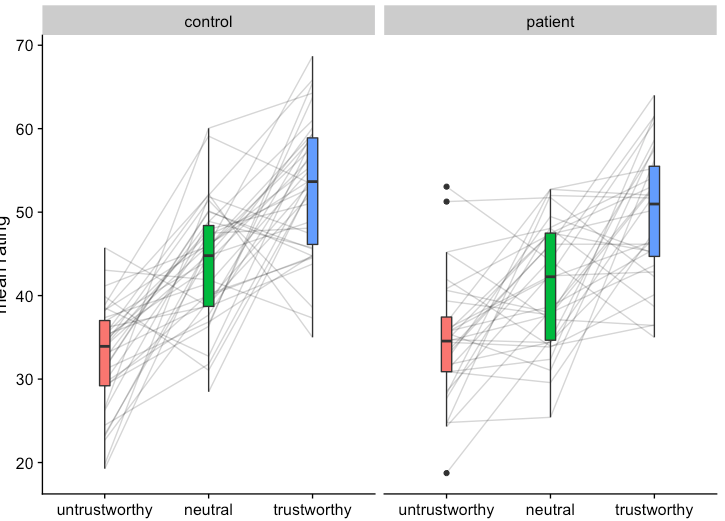


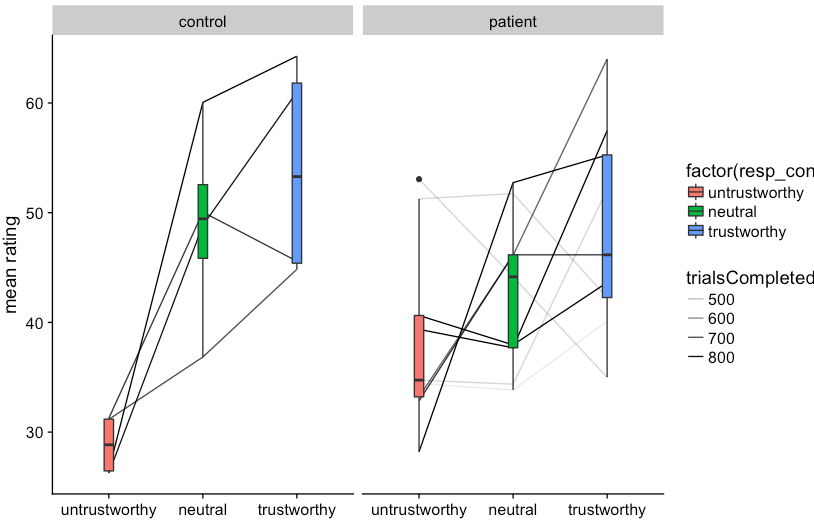


**Supplemental Figure 1, related to Figure 2C in the main text:** Exploratory analyses including the thirteen participants that did not complete all trials of the reverse correlation task. Top panel shows the ratings of 205 images from all 70 participants (56 full datasets, 13 incomplete datasets, same conventions as Figure 3 in the main text). Bottom panel shows ratings of the thirteen participants with incomplete datasets (39 images). In the bottom panel, the shade of grey represents the number of completed trials in the reverse correlation task (darker shades equal more completed trials, range: 404 to 824 of the 900 trials completed). The Inclusion of the 13 participants with incomplete datasets did not alter any of the main findings (main effect of ‘response category’: F(2, 126) = 77.640, p < .001, partial η^2^ = .55; main effect of ‘participant group’: F(1, 63) = 1.700, p = .197, partial η^2^ = .026; interaction of ‘response category’ and ‘participant group’: (F(2, 126) = 2.440, p = .091, partial η^2^ = .037).
